# Serum levels of 14-3-3η protein supplement C-reactive protein and rheumatoid arthritis-associated antibodies to predict clinical and radiographic outcomes in a prospective cohort of patients with recent-onset inflammatory polyarthritis

**DOI:** 10.1186/s13075-016-0935-z

**Published:** 2016-02-01

**Authors:** Nathalie Carrier, Anthony Marotta, Artur J. de Brum-Fernandes, Patrick Liang, Ariel Masetto, Henri A. Ménard, Walter P. Maksymowych, Gilles Boire

**Affiliations:** Centre Hospitalier Universitaire de Sherbrooke, Sherbrooke, QC Canada; Augurex Life Sciences Corp, Vancouver, BC Canada; Université de Sherbrooke, Sherbrooke, QC Canada; Research Institute of the McGill University Health Center, Montreal, QC Canada; University of Alberta, Edmonton, AB Canada; Division of Rheumatology, CHUS-Fleurimont, 3001, 12th Avenue North, Room 3853, Sherbrooke, QC J1H 5N4 Canada

**Keywords:** Recent-onset inflammatory arthritis, 14-3-3η, Radiographic progression, Anti-CCP2 antibodies, Anti-Sa/citrullinated vimentin antibodies, Rheumatoid factor, CRP, SDAI remission

## Abstract

**Background:**

Age, C-Reactive Protein (CRP) and autoantibodies (Abs) are associated with worse prognosis in patients with recent-onset inflammatory polyarthritis (EPA). Serum 14-3-3η protein is a joint-derived biomarker that up-regulates cytokines and enzymes that perpetuate local and systemic inflammation and may contribute to joint damage. Our objective was to evaluate, over a 5-year prospective period of observation, the additional prognostic potential of serum 14-3-3η protein in EPA patients.

**Methods:**

Clinical variables, serum and radiographs (scored according to the Sharp/van der Heijde (SvH) method) were collected serially. Relationships between serum 14-3-3η protein and other biomarkers were computed with Spearman correlations. Outcomes were Simple Disease Activity Index (SDAI) scores and joint damage progression: ΔSvH for SvH score and ΔErosion for its Erosive component. The additional predictive contribution of 14-3-3η was defined using generalized estimating equations (GEE) and generalized linear mixed models (GLMM).

**Results:**

Among 331 patients, baseline 14-3-3η was ≥0.19 and ≥0.50 ng/ml in 153 (46.2 %) and 119 (36.0 %), respectively; CRP was >8.0 mg/L in 207 (62.5 %), and at least one Ab (Rheumatoid Factor, anti-CCP2 or anti-Sa/citrullinated vimentin) was positive in 170 (51.5 %). Elevated 14-3-3η levels moderately correlated with positive Abs, but not with elevated CRP. Baseline 14-3-3η ≥0.19 ng/ml was associated with more radiographic progression over 5 years. The optimal levels of baseline 14-3-3η to predict radiographic progression was defined by ROC curves at 0.50 ng/ml. Levels of 14-3-3η ≥0.50 ng/ml at baseline were associated with lower likelihoods of ever reaching SDAI remission (RR 0.79 (95 % CI 0.64–0.98), p = 0.03) and higher subsequent progression of Total and Erosion SvH scores. Elevated levels of 14-3-3η during follow-up also predicted higher subsequent progression, even in patients in SDAI remission. Decreases of 14-3-3η levels by at least 0.76 ng/ml and reversion to negative during follow-up associated with less subsequent radiographic progression. In multivariate models, elevated 14-3-3η interacted with positive Abs, elevated CRP and older age to predict subsequent radiographic progression.

**Conclusions:**

Levels of 14-3-3η protein ≥0.50 ng/ml predict poorer clinical and radiographic outcomes in EPA, both at baseline and after initiation of treatment, even in SDAI remitters. 14-3-3η, CRP, age and Abs represent independent predictors of subsequent joint damage.

**Trial registration:**

ClinicalTrials.gov ID: NCT00512239. Registered August 6, 2007.

**Electronic supplementary material:**

The online version of this article (doi:10.1186/s13075-016-0935-z) contains supplementary material, which is available to authorized users.

## Background

Rheumatoid arthritis (RA) is the most prevalent chronic inflammatory joint disease [1]. Despite recent advances in treatment, RA remains a significant cause of morbidity, invalidity and premature mortality [2]. The concept of treat-to-target (T2T) strategies tailored to individual patients defines specific clinical targets for disease activity (e.g., remission or at least low disease activity), reduction of C-reactive protein (CRP) and/or erythrocyte sedimentation rate (ESR) to normal values and the halting of joint damage within a reasonable time frame. T2T approaches are particularly effective in early disease to prevent joint destruction and increase the likelihood of achieving remission, giving rise to the concept of a ‘window of opportunity’ [2]. One of the limitations to this approach is the inability of currently available biomarkers to identify many RA patients early with recent-onset polyarthritis (EPA), such that early referral by primary care and effective triaging by joint specialists remains incomplete [3]. A second limitation resides in the inability of the same biomarkers to determine the expected outcomes at the individual level, which would allow immediate adjustment of treatment selection and intensity to the severity of the disease [4].

The 14-3-3 family of conserved regulatory proteins consists of seven isoforms: α/β, γ, δ/ζ, ε, η, θ/τ and σ [5]. These proteins normally exist as ubiquitous intracellular adapters (or chaperones) that interact with over 200 intracellular proteins and contribute to the modulation of their activities. 14-3-3η is detectable at significantly higher levels in the serum and synovial fluid from patients with RA when compared with healthy subjects and individuals with other autoimmune conditions and viral/bacterial infections [6]. Extracellular 14-3-3η at concentrations detectable in RA patient serum acts as a cell damage signal that potently induces pro-inflammatory cytokines and bone-degrading enzymes [7].

We report the use of serial measurements of serum 14-3-3η protein to evaluate its additive role as a diagnostic aid in early disease and as a predictive biomarker for more severe outcomes of RA at the clinical and radiographic levels. We also evaluated its additive prognostic potential in combination with established biomarkers in clinical use, such as CRP and RA-associated autoantibodies.

## Methods

### Early undifferentiated polyarthritis (EUPA) patient cohort

The longitudinal EUPA cohort was previously described [8–10]. Starting in 1998, consecutive adult patients with at least three swollen joints for one to twelve months were evaluated at the Centre hospitalier universitaire de Sherbrooke (CHUS) and asked to participate. We excluded patients with bacterial or crystal-induced arthritis or a defined connective tissue disease or systemic vasculitis according to American College of Rheumatology (ACR) criteria [11]. Patients were treated by rheumatologists using early and intensive treatment with dosage individualized to achieve sustained remission defined as 0 swollen joints out of 66 joints [2, 12]. Serum samples were coded and stored at –20 °C. The Ethics Review Board of the CHUS approved the study (ClinicalTrials.gov ID: NCT00512239) and all patients provided written informed consent.

### Disease variables

A rheumatologist completed joint counts and a trained coordinator conducted a structured interview at inclusion and at each of the follow-up visits scheduled at 18, 30, 42 and 60 months after onset. Time of onset was self-reported as the week during which symptoms/signs of inflammatory arthropathy had appeared. Variables assessed included demographics; 68 tender joint count (TJC) and 66 swollen joint count (SJC); drug use at and between each visit; modified Health Assessment Questionnaire (M-HAQ) [13]; serum CRP (upper normal limit: 8.0 mg/L); components of the Simplified Disease Activity Index (SDAI); and RA-associated antibodies (see below). Radiographs of the hands and feet were obtained at inclusion and at each scheduled assessment. Joint space narrowing and erosions were scored on these radiographs according to the Sharp score modified by van der Heijde (SvH) method, with a maximum score of 448 units [14]. Radiographs were read in known time sequence by two blinded assessors, one of which was a study investigator (GB). Under these conditions, the smallest detectable change (SDC) was 5 units [15].

### RA-associated antibodies

IgM rheumatoid factor (RF) was measured using RapiTex RF (Dade Behring Inc, Newark DE, USA) (positive ≥40 IU/ml). Anti-CCP2 antibodies were measured using QuantaLite™, Inova Diagnostics (San Diego, CA, USA) using titer levels as suggested by the manufacturer (positive >20.0 U/ml) or, since 2009, using the EuroImmun assay (positive >5.0 U/ml). As both the EuroImmun and the Inova assays use the same enzyme linked immunosorbent assay (ELISA) plates coated with the same antigens (as do all other antibodies to cyclic citrullinated peptide, second generation (anti-CCP2) assays across the world), have similar sensitivity and specificity, and give linear results across a wide range of concentrations, their results are easily interconvertible using the following logarithmic transformation:

Inova anti-CCP2 = –26.44 + 28.86 * ln(EuroImmun anti-CCP2).

The anti-Sa/citrullinated vimentin in-house ELISA was described previously (positive threshold ≥0.20 optical density units) [16]. From 2012 on, we used a commercial anti-Sa assay (EuroImmun; positive >19 RU/ml), validated against our in-house ELISA [17].

### Outcomes

Radiographic progression was defined by the difference between the SvH scores over time and baseline damage. Definite SvH progression was defined as an increase ≥5 in either the total score (ΔSvH) or its erosion component (ΔErosion). Remission was defined as SDAI ≤3.3 [18].

### Serum 14-3-3η measurements

Serum 14-3-3η levels were measured using the 14-3-3η ELISA according to the manufacturer’s protocol (Augurex Life Sciences Corp, Vancouver, BC, Canada). Samples with levels below the reportable range were assigned a concentration of 0.0 ng/ml and those with levels above the upper limit were defined as having levels ≥20 ng/ml. Positivity for 14-3-3η was defined by the manufacturer at ≥0.19 ng/ml.

### Statistical methods

Quantitative variables were presented as mean (standard deviation (SD)) or as the median and 25th–75th percentiles (interquartile range (IQR)). Categorical variables were presented with frequencies and percentages. 14-3-3η levels at each visit were compared to baseline levels using the Wilcoxon sign rank test. SDAI scores and remission status, SvH score, ΔSvH and ΔErosion were compared with baseline 14-3-3η positivity using the Mann–Whitney *U* test, independent samples Student *t* test and Pearson’s chi-square test, as appropriate. To evaluate the benefit of using positive 14-3-3η protein to support RA diagnosis among EPA patients, we combined elevated 14-3-3η levels with positive RA-associated antibodies. The incremental benefit of positive 14-3-3η over single types of antibody or combinations of antibodies was calculated as the patients identified by a positive 14-3-3η result among patients negative for the biomarker of reference, divided by the number of patients positive for the biomarker of reference. The significance of the observed incremental benefit was evaluated using the McNemar test. Receiver operator characteristic (ROC) curves were used to establish the optimal threshold of baseline 14-3-3η positivity for prediction of ΔSvH ≥5 from inclusion to 5 years. Spearman correlation was used to evaluate association between baseline variables. Generalized linear mixed (GLMM) models with repeated measures were used to evaluate effect of baseline 14-3-3η positivity on SDAI, SvH score, ΔSvH and ΔErosion over time. Generalized estimating equations (GEE) for binary outcomes with repeated measures were used to measure relative risk (RR) of attaining SDAI remission, ΔSvH ≥5, ΔErosion ≥5 and use of biologic DMARDs over time according to 14-3-3η positivity at inclusion or at the previous visit. Multivariate GLMM and GEE models were computed to evaluate which baseline variables explained progression of SvH score over time. Age, gender, 14-3-3η, antibodies and CRP were analyzed as continuous or categorical variables. In analyses with repeated measures, the covariance structure (autoregressive, compound symmetry, variance components or unstructured) with the lowest Akaike information criteria (AIC) for GLMM or quasi-AIC (QIC) for GEE was used to model the subject variation. All variables and interaction terms were included in the first model. We then deleted one by one the variables or interaction terms that were not significant; when deletion of a variable or interaction term resulted in an increase rather than a decrease in the AIC or QIC, this variable or interaction term was kept in the model. This process continued until we reached the smallest AIC or QIC. All analyses were based only on available data without imputation, as fewer than 5 % of values for each variable were missing. Statistical analysis was performed using SAS software version 9.3, SPSS software version 23.0 and GraphPad Prism Software version 6.00 for Windows. A *p* value <0.05 denoted statistical significance.

## Results

### Elevated 14-3-3η serum protein levels at baseline and over time

As of 15 May 2014, from the 688 included in the ongoing EUPA cohort, 331 patients (62 % women, mean age 60 years) had completed 5 years of follow up and were selected for this study (Table 1). Median symptom duration at baseline was 3 months, and over 92 % already fulfilled either the 1987 ACR or the 2010 ACR/EULAR classification criteria for RA. We previously reported that not meeting the 1987 ACR criteria for RA at baseline did not significantly impact subsequent outcomes in our patients [10, 16]. Disease activity was moderate to high: median (IQR) SDAI 30.1 (19.8–45.2); median M-HAQ (IQR) 0.8 (0.4-1.4). Baseline joint damage was low, with median total SvH (IQR) of 2 (0–6) and median erosion SvH (IQR) of 1 (0–3). Patients rapidly received DMARDs, usually methotrexate (MTX), alone or in combination with other DMARDs [8–10, 16].

**Table 1:** Baseline cohort characteristics

Variable	Number	Value
Age, median (IQR^a^), years	331	60 (49.4–68.6)
Age ≥65 years, n (%)	331	122 (36.9)
Women, n (%)	331	205 (61.9)
Current smoker, n (%)	330	69 (20.9)
Symptom duration, median (IQR), months	331	3.1 (1.7–5.7)
Body mass index, median (IQR), kg/m^2^	309	26.1 (23–29.8)
25–29.9, n (%)		104 (33.7)
≥30, n (%)		74 (23.9)
Swollen joint count, 28 joints, median (IQR)	330	9 (5–15)
Tender joint count, 28 joints, median (IQR)	329	9 (4–16)
Fulfilling 1987 ACR criteria for rheumatoid arthritis, n (%)	331	275 (83.1)
Fulfilling 2010 EULAR/ACR criteria for rheumatoid arthritis, n (%)	330	281 (85.2)
Fulfilling 1987 or 2010 sets of criteria for rheumatoid arthritis, n (%)	330	304 (92.1)
Disease Activity Score, 28 joints-C-reactive protein, median (IQR)	328	5.1 (4.2–6.2)
Simplified Disease Activity Index, median (IQR)	328	30.1 (19.8–45.2)
Modified Health Assessment Questionnaire, median (IQR)	329	0.8 (0.4–1.4)
Total SvH score, median (IQR)	328	2 (0–6)
SvH erosion score, median (IQR)	328	1 (0–3)
Erythrocyte sedimentation rate, mm/h, median (IQR)	331	33 (17–46)
C-reactive protein, mg/L, median (IQR)	331	13.1 (4.5–32.0)
C-reactive protein >8.0 mg/L	331	207 (62.5)
Rheumatoid factor-positive, ≥40 IU/ml	331	146 (44.1)
Anti-CCP2 positive	331	133 (40.2)
Anti-Sa positive	331	73 (22.1)
14-3-3η, ng/ml, median (IQR)	331	0.1 (0.0–1.9)
14-3-3η positive, ≥0.19 ng/ml	331	153 (46.2)
14-3-3η positive, ≥0.50 ng/ml	331	119 (36.0)

^a^ *IQR* 25th–75th percentiles. *ACR* American College of Rheumatology. *EULAR* European League Against Rheumatism, *Anti-CCP2* antibodies to citrullinated peptides, second generation. *SvH* Sharp score modified by van der Heijde

Baseline levels of 14-3-3η protein were ≥0.19 ng/ml (threshold suggested by the manufacturer) in 153 patients (46.2 %) and ≥0.50 ng/ml (the optimal prognostic threshold defined in our cohort; see below) in 119 patients (36.0 %). CRP was >8.0 mg/ml in 207 patients (62.5 %), and RF, anti-CCP2 and anti-Sa antibodies (Abs) were positive in 146 (44.1 %), 133 (40.2 %) and 73 (22.1 %) of patients, respectively; 170 patients (51.5 %) had at least one positive antibody.

Median (IQR) 14-3-3η levels significantly decreased between baseline (0.14 (0.03–1.86) ng/ml) and each follow-up measurement: 0.11 (0.03–1.12), *p* <0.0001; 0.11 (0.03–0.99), *p* <0.0001; 0.10 (0.02–0.93), *p* <0.0001; and 0.13 (0.02–1.14) ng/ml, *p* = 0.001 at 18, 30, 42 and 60 months, respectively (Additional file 1). The mean (+/- SD) decrease in 14-3-3η titers between baseline and subsequent visits was 0.72 (5.20), 0.62 (5.98), 0.86 (5.54) and 0.58 (6.11) ng/ml at 18, 30, 42 and 60 months, respectively. As a consequence, the proportion of patients remaining 14-3-3η-positive among those with baseline levels ≥0.50 ng/ml dropped to 70 % and 75 % at 42 and 60 months, respectively (Additional file 1). On the contrary, 90–95 % of the patients with baseline 14-3-3η <0.50 ng/ml remained negative over follow up (Additional file 1).

### Complementarity of 14-3-3η in identification of patients with early RA

To assess the potential of 14-3-3η assessment to help primary care providers consider RA diagnosis in EPA patients, we combined elevated 14-3-3η levels with positive RA-associated antibodies. Using the manufacturer’s suggested positivity cutoff of ≥0.19 ng/ml increased the number of patients with at least one of the four biomarkers to 194 (58.8 %), a 14.1 % incremental benefit (Table 2). Of the 24 additional patients identified by elevated 14-3-3η, 20 already fulfilled 1987 and/or 2010 criteria for RA at baseline, despite being antibody-negative.

**Table 2:** Additional contribution of positive 14-3-3η to identify rheumatoid arthritis amongst patients with early inflammatory arthritis

	1987+/2010+	1987+/2010–	1987–/2010+	1987–/20–	Total	Incremental benefit of 14-3-3η
	(n = 252) number (%)	(n = 23) number (%)	(n = 29) number (%)	(n = 26) number (%)	(n = 330^a^) number (%)	
Rheumatoid factor+	140 (55.6)	0 (0)	6 (20.7)	0 (0)	146 (44.2)	NA
Anti-CCP2+	119 (47.2)	0 (0)	14 (48.3)	0 (0)	133 (40.3)	NA
Anti-Sa+	67 (26.6)	0 (0)	6 (20.7)	0 (0)	73 (22.1)	NA
Rheumatoid factor + and/or anti-CCP2+	153 (60.7)	0 (0)	14 (48.3)	0 (0)	167 (50.6)	NA
Rheumatoid factor + and/or anti-CCP2+ and/or anti-Sa+	155 (61.5)	0 (0)	15 (51.7)	0 (0)	170 (51.5)	NA
14-3-3η ≥0.19 ng/ml	134 (50.6)	1 (10)	14 (43.8)	4 (17.4)	153 (46.4)	NA
14-3-3η ≥0.50 ng/ml	111 (44.0)	0 (0)	8 (27.6)	0 (0)	119 (36.1)	NA
14-3-3η ≥ 0.19 ng/ml and/or						
Rheumatoid factor+	161 (63.9)	1 (4.3)	15 (51.7)	4 (15.4)	181 (54.8)	24.0 %***
Anti-CCP2+	158 (62.7)	1 (4.3)	19 (65.5)	4 (15.4)	182 (55.2)	36.8 %***
Anti-Sa+	144 (57.1)	1 (4.3)	15 (51.7)	4 (15.4)	164 (49.7)	124.7 %***
Rheumatoid factor + and/or anti-CCP2+	168 (66.7)	1 (4.3)	19 (65.5)	4 (15.4)	192 (58.2)	15.0 %***
Rheumatoid factor + and/or anti-CCP2+ and/or anti-Sa+	170 (64.2)	1 (10)	19 (59.4)	4 (17.4)	194 (58.8)	14.1 %***
14-3-3η ≥ 0.50 ng/ml and/or						
Rheumatoid factor+	150 (59.5)	0 (0)	9 (31.0)	0 (0)	159 (48.2)	8.9 %***
Anti-CCP2+	147 (58.3)	0 (0)	15 (51.7)	0 (0)	162 (49.1)	21.8 %***
Anti-Sa+	126 (50.0)	0 (0)	11 (37.9)	0 (0)	137 (41.5)	87.7 %***
Rheumatoid factor + and/or anti-CCP2+	159 (63.1)	0 (0)	15 (51.7)	0 (0)	174 (52.7)	4.2 %**
Rheumatoid factor + and/or anti-CCP2+ and/or anti-Sa+	161 (63.9)	0 (0)	16 (55.2)	0 (0)	177 (53.6)	4.1 %**

^a^Data insufficient to assess 2010 American College of Rheumatology/European League Against Rheumatism criteria in one patient; **p <0.01; ***p <0.001. *Anti CCP2* antibodies to citrullinated peptides, second generation. *NA*Not applicable

### Defining the optimal 14-3-3η threshold to predict worse radiographic outcomes

The higher the 14-3-3η baseline levels the stronger the association with radiographic progression between baseline and each of the follow-up evaluations (*r* approximately 0.19, *p* <0.001). Similarly, the higher the decrease in 14-3-3η titers between baseline and 18 months, the lesser the radiographic progression from 18 to 30 months (*r* = –0.14, *p* = 0.018); ROC curve analyses defined a decrease of <0.76 ng/ml as the best to predict definite radiographic progression (ΔSvH ≥5) over 5 years: 28.6 % vs 14.3 %, RR (95 % CI) = 2.00 (1.20–3.34), *p* = 0.01 (area under the curve (AUC) = 0.567, sensitivity = 0.844, specificity = 0.308). The 21 patients whose 14-3-3η status reverted from ≥0.19 at baseline to negative by 18 months had lower median (IQR) baseline titers: 0.3 (0.3–0.6) versus 3.6 (1.0–21.0) ng/ml in those who remained positive. As low or transiently positive titers of a biomarker are usually less specific and have weaker associations with specific outcomes, ROC curves were drawn to define the optimal level of baseline 14-3-3η to predict radiographic outcomes. Using two thresholds for ΔSvH or ΔErosion (3 or 5 units) and the shortest length of observation (3 years), the optimal level of baseline 14-3-3η was 0.490 ng/ml (ROC curves not shown). As no patient had values between 0.490 and 0.500, we therefore selected 0.50 ng/ml as the optimal prognostic threshold for 14-3-3η. The threshold of 0.50 ng/ml at baseline was associated with more erosive progression (ΔErosion ≥5) over 5 years (GEE: RR (95 % CI) = 2.01 (1.49-2.72), *p* <0.001) compared to levels <0.19 ng/ml (Table 3). The 31 patients with baseline 14-3-3η levels between 0.19 and 0.49 ng/ml and the 169 patients with levels <0.19 ng/ml had similar risk for total SvH and erosion progression (Table 3). Compared to those with 14-3-3η ≥0.19 but <0.50 ng/ml, patients with levels ≥0.50 ng/ml had higher risk of definite radiographic progression (ΔSvH ≥5; RR 1.82 (1.11–3.00), *p* <0.05) and erosive progression (ΔErosion ≥5; RR 2.15 (1.21–3.83), *p* <0.01). As a consequence, a threshold for positivity of ≥0.50 ng/ml was selected for presentation in this report. Results of some analyses using a threshold of ≥0.19 ng/ml are shown in Additional file 2.

**Table 3:** Impact of biomarkers on radiographic and erosive progression (ΔSvH ≥5 and ΔErosion ≥5)

		ΔSvH ≥ 5		ΔErosion ≥ 5	
Baseline variables	Total, number	at 60 m, number (%)	RR (95 % CI) from GEE over time	at 60 m, number (%)	RR (95 % CI) from GEE over time
14-3-3η, ng/ml					
<0.19	169	73 (43.2)	1	48 (28.4)	1
0.19–0.50	31	12 (38.7)	0.86 (0.52–1.43)	10 (32.3)	0.93 (0.51–1.70)
≥0.50	115	74 (64.3)	1.57 (1.24–1.97) ***	60 (52.2)	2.01 (1.49–2.72) ***
14-3-3η, ng/ml					
<0.19	169	73 (43.2)	1	48 (28.4)	1
≥0.19	146	86 (58.9)	1.40 (1.11–1.77) **	70 (47.9)	1.77 (1.31–2.38) ***
14-3-3η, ng/ml					
<0.50	200	85 (42.5)	1	58 (29.0)	1
≥0.50	115	74 (64.3)	1.60 (1.28–2.00) ***	60 (52.2)	2.04 (1.53–2.70) ***
Age, years					
<65	198	81 (40.9)	1	62 (31.3)	1
≥65	117	78 (66.7)	1.71 (1.36–2.13) ***	56 (47.9)	1.51 (1.13–2.01) **
C-reactive protein, mg/L					
≤8.0	117	51 (43.6)	1	30 (25.6)	1
>8.0	198	108 (54.5)	1.46 (1.12–1.90) **	88 (44.4)	1.87 (1.31–2.66) ***
Anti-CCP2 status					
Negative	190	85 (44.7)	1	59 (31.1)	1
Positive	125	74 (59.2)	1.44 (1.14–1.80) **	59 (47.2)	1.74 (1.31–2.33) ***
RF ≥40 IU/ml					
Negative	176	76 (43.2)	1	51 (29.0)	1
Positive	139	83 (59.7)	1.51 (1.20–1.90) ***	67 (48.2)	2.02 (1.50–2.72) ***
Anti-Sa status					
Negative	246	110 (44.7)	1	76 (30.9)	1
Positive	69	49 (71.0)	1.72 (1.38–2.14) ***	42 (60.9)	2.22 (1.69–2.92) ***
RF or anti-CCP2 positive					
Negative	157	67 (42.7)	1	44 (28.0)	1
Positive	158	92 (58.2)	1.53 (1.21–1.94) ***	74 (46.8)	2.02 (1.47–2.76) ***
14-3-3η ≥0.50 ng/ml and age ≥65 years					
Both negative	117	34 (29.1)	1	24 (20.5)	1
One positive	164	98 (59.8)	2.35 (1.71–3.23) ***	72 (43.9)	2.40 (1.61–3.57) ***
Both positive	34	27 (79.4)	2.96 (2.07–4.24) ***	22 (64.7)	3.46 (2.21–5.42) ***
14-3-3η ≥0.50 and C-reactive protein >8.0 mg/L					
Both negative	73	28 (38.4)	1	16 (21.9)	1
One positive	171	80 (46.8)	1.25 (0.88–1.76)	56 (32.7)	1.58 (0.97–2.58)
Both positive	71	51 (71.8)	2.21 (1.58–3.09) ***	46 (64.8)	3.48 (2.17–5.56) ***
14-3-3η ≥0.50 ng/ml and anti-CCP2					
Both negative	163	68 (41.7)	1	45 (27.6)	1
One positive	64	34 (53.1)	1.4 (1.04–1.89) *	27 (42.2)	1.74 (1.19–2.54) **
Both positive	88	57 (64.8)	1.7 (1.32–2.20) ***	46 (52.3)	2.27 (1.62–3.16) ***
14-3-3η ≥0.50 ng/ml and RF					
Both negative	165	69 (41.8)	1	44 (26.7)	1
One positive	46	23 (50.0)	1.31 (0.93–1.86)	21 (45.7)	1.80 (1.19–2.72) **
Both positive	104	67 (64.4)	1.68 (1.32–2.15) ***	53 (51.0)	2.32 (1.67–3.22) ***
14-3-3η ≥ 0.50 ng/ml and anti-Sa					
Both negative	185	75 (40.5)	1	51 (27.6)	1
One positive	76	45 (59.2)	1.36 (1.03–1.79) *	32 (42.1)	1.64 (1.15–2.34) **
Both positive	54	39 (72.2)	2.02 (1.57–2.59) ***	35 (64.8)	2.80 (2.04–3.85) ***
14-3-3η ≥ 0.50, “RF and/or anti-CCP2”					
Both negative	152	64 (42.1)	1	41 (27)	1
One positive	110	71 (64.5)	1.23 (0.87–1.74)	57 (51.8)	1.59 (1.04–2.44) *
Both positive	53	24 (45.3)	1.71 (1.33–2.19) ***	20 (37.7)	2.34 (1.67–3.27) ***
14-3-3η ≥ 0.50, Age ≥65, C-reactive protein >8.0					
All negative	43	11 (25.6)	1	7 (16.3)	1
Only C-reactive protein positive	74	23 (31.1)	1.25 (0.67–2.32)	17 (23.0)	1.54 (0.71–3.36)
Only 14-3-3η positive	34	16 (47.1)	1.87 (0.97–3.60)	10 (29.4)	2.18 (0.94–5.06)
Only age positive	30	17 (56.7)	2.41 (1.31–4.43) **	9 (30.0)	2.06 (0.86–4.89)
Only C-reactive protein and age positive	53	34 (64.2)	2.97 (1.71–5.18) ***	25 (47.2)	3.27 (1.59–6.71) **
Only 14-3-3η and C-reactive protein- positive	47	31 (66.0)	3.21 (1.85–5.59) ***	28 (59.6)	4.65 (2.33–9.29) ***
Only 14-3-3η and age-positive	10	7 (70.0)	2.22 (1.02–4.84) *	4 (40.0)	2.47 (0.85–7.17)
All positive	24	20 (83.3)	3.90 (2.25–6.78) ***	18 (75.0)	5.49 (2.73–11.08) ***

*p <0.05, **p <0.01, ***p <0.001. *ΔSvH* progression in the total Sharp score modified by van der Heijde, *ΔErosion* progression in the erosion component of the Sharp score modified by van der Heijde, *GEE* general estimating equation, *RF* rheumatoid factor, *Anti-CCP2* antibodies to citrullinated peptides, second generation

### Baseline 14-3-3η serum protein levels identify patients with refractory disease over 5 years

**Fig. 1:**
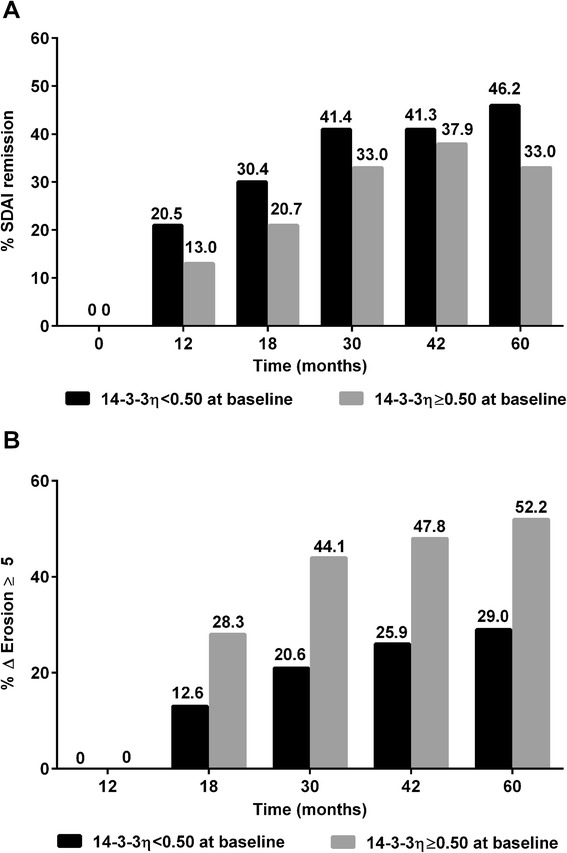
Simplified Disease Activity Index (*SDAI*) remission (**a**) and erosive progression (**b**) over 5 years according to baseline 14-3-3η positivity. General estimating equations analysis was performed to compare SDAI scores and radiographic progression over time with baseline 14-3-3η ≥0.50 ng/ml. **a** SDAI remission over time was significantly lower in patients with baseline 14-3-3η ≥0.50 (relative risk (RR) = 0.79 (95 % CI 0.64–0.98), *p* = 0.03). **b** Definite erosive progression (*ΔErosion ≥5*) over time was significantly higher in patients with baseline 14-3-3η ≥0.50 ng/ml (RR = 2.04 (95 % CI 1.53–2.70), *p* <0.001)

At baseline, median SDAI, DAS28-CRP, and total and erosion SvH levels were not significantly different based on positive or negative 14-3-3η levels ≥0.50 ng/ml; similar results were obtained with a threshold ≥0.19 (data not shown). When patients were grouped according to 14-3-3η-positive status at baseline, GLM analysis revealed that SDAI scores were significantly higher (*p* = 0.002) across all visits in 14-3-3η-positive patients at baseline (data not shown). GEE analysis revealed that fewer patients achieved SDAI remission based on a baseline 14-3-3η ≥0.50, delivering a relative risk (RR) of 0.79 (95 % CI 0.64–0.98), *p* = 0.03 (Fig. 1a); similar results were obtained with the 0.19 ng/ml threshold (Additional file 2A). Not surprisingly, baseline 14-3-3η ≥0.50 ng/ml was also associated with an increased risk of definite radiographic progression (ΔSvH ≥5; RR =1.60 (95 % CI 1.28–2.00), *p* <0.001; ΔErosion ≥5; RR =2.04 (95 % CI 1.53–2.70), *p* <0.001 (Fig. 1b), and a trend for use of biologic DMARDs over time (RR =1.62 (0.94–2.80), *p* = 0.085). Baseline 14-3-3η ≥0.19 ng/ml was associated with a lower risk for definite radiographic progression (ΔSvH ≥5; RR =1.39 (95 % CI 1.11–1.74), *p* = 0.005 (Additional file 2B). As a consequence, patients with baseline 14-3-3η ≥0.50 ng/ml had higher mean radiographic progression at 60 months (mean ± SD for ΔSvH: 13.08 ± 17.34 vs 7.30 ± 13.38, *p* = 0.002 and ΔErosion: 9.23 ± 13.22 vs 4.37 ± 9.05, *p* = 0.001).

### Elevated 14-3-3η protein levels during follow up predict more rapid radiographic progression over the following years, even in patients in SDAI remission

**Fig. 2:**
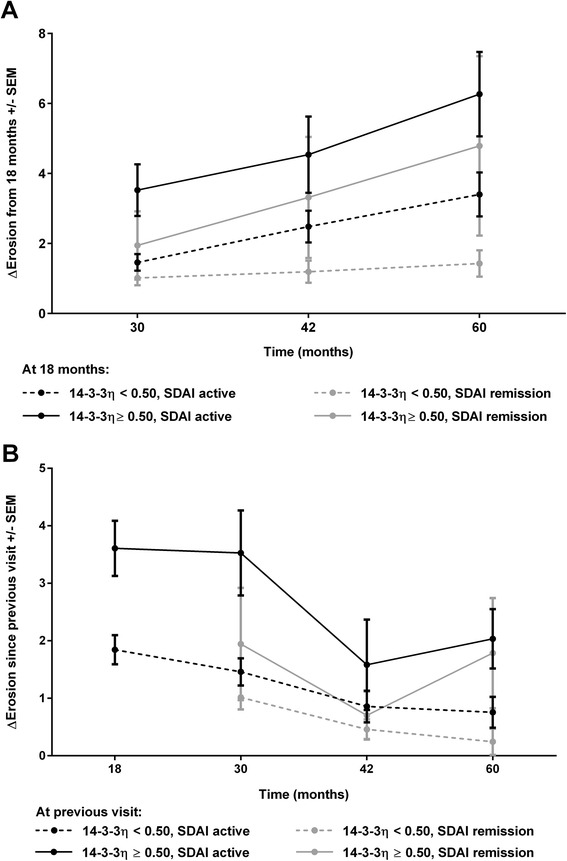
Radiographic progression over 5 years according to Simplified Disease Activity Index (*SDAI*) remission and 14-3-3η positivity. Generalized linear model analysis was performed to compare radiographic progression over time with 14-3-3η ≥0.50 ng/ml. **a** Relative to patients with lower 14-3-3η levels, erosive damage progression (*ΔErosion*) was significantly higher in patients with 14-3-3η ≥0.50 ng/ml at 18 months, whether the patients were in SDAI remission (*p* = 0.002) or had active SDAI levels at 18 months (*p* <0.001). **b** Relative to patients with lower 14-3-3η levels, yearly erosive damage progression (*ΔErosion*) was significantly higher in patients with 14-3-3η ≥0.50 ng/ml at the previous visit, whether the patients were already in SDAI remission (*p* = 0.0079) or had active SDAI levels (*p* <0.001) at the previous visit. *SEM* standard error of the mean

Only 308/331 patients had a full series of baseline, 18-month and 30-month radiographs; the 23 without either 18-month or 30-month radiographs had similar 14-3-3η levels at baseline and at 30 months. Of the 308 patients with complete radiographs, 84 (27.3 %) were 14-3-3η-positive (≥0.50) at both baseline and 18-month visits, and 23 patients (7.5 %), who were positive at baseline, reverted to negative and 11 patients (3.6 %), who were initially negative converted to positive at 18 months. The 23 patients who sero-reverted from positive 14-3-3η to negative at 18 months had mild progression between 18 and 30 months, similar to the 190 persistently negative patients (1.77 ± 3.83 vs 2.74 ± 3.02, *p* = 0.243). On the contrary, patients who were 14-3-3η-positive at 18 months developed significantly higher ΔSvH over the following years, both in those with active disease as assessed by the SDAI at 18 months and to a lesser extent, in patients in SDAI remission at 18 months (Fig. 2a). Radiographic progression slowed but persisted after the 30-month visit, despite a gradual increase in use of biologic DMARDs (6.4 %, 11.6 %, 13.8 % and 14.8 % at 18, 30, 42 and 60 months, respectively) and in the prevalence of SDAI remission (Fig. 1a). Nonetheless, 14-3-3η ≥0.50 ng/ml at each visit correlated with more rapid progression over the following years, even in those who were in SDAI remission at the same visit (Fig. 2b).

### Elevated 14-3-3η serum levels improve outcome prediction associated with positive RA-associated antibodies, CRP and older age

Before increasing the range of available biomarkers used for outcome prediction in RA, we need to estimate the additional information contributed by 14-3-3η detection. Table 4 presents the Spearman correlation between baseline variables. As expected, antibodies correlated moderately/strongly with each other. Interestingly, positive antibodies and 14-3-3η ≥0.50 ng/ml were also moderately correlated (*r* = 0.679 for RF, 0.539 for anti-CCP2 and 0.437 for anti-Sa). Age was moderately correlated with SvH scores, and 14-3-3η levels negatively correlated weakly with age (*r* = -0.140, *p* = 0.01). Levels of CRP and 14-3-3η did not correlate (Spearman correlation *r* =0.001; *p* = 0.996) (Table 4).

**Table 4:** Spearman correlation (*ρ*) between baseline continuous and categorical variables

Baseline variables	14-3-3η	14-3-3η	14-3-3η	Anti-CCP2	Anti-CCP2-positive	RF	RF positive	Age (years)	Age ≥65 years	BMI ≥30
		≥0.19	≥0.50							
14-3-3η	1									
14-3-3η ≥ 0.19	0.865 ***	1								
14-3-3η ≥ 0.50	0.833 ***	0.808 ***	1							
Anti-CCP2	0.529 ***	0.515 ***	0.530 ***	1						
Anti-CCP2-positive	0.529 ***	0.524 ***	0.539 ***	0.849 ***	1					
RF	0.716 ***	0.659 ***	0.731 ***	0.624 ***	0.638 ***	1				
RF positive	0.669 ***	0.616 ***	0.679 ***	0.616 ***	0.661 ***	0.927 ***	1			
Anti-Sa positive	0.409 ***	0.413 ***	0.437 ***	0.527 ***	0.519 ***	0.500 ***	0.481 ***			
Age (years)	-0.140 *	-0.118 *	-0.087	-0.033	-0.085	-0.069	-0.104	1		
Age ≥65 years	-0.151 **	-0.131 *	-0.103	-0.040	-0.095	-0.071	-0.099	0.836 ***	1	
BMI ≥30	-0.028	0.042	0.007	-0.031	-0.013	-0.014	-0.039	-0.06	-0.024	1
Gender (women vs men)	-0.031	-0.034	-0.074	-0.010	-0.033	-0.044	-0.030	-0.158 **	-0.072	0.000
Symptom duration (months)	0.119 *	0.127 *	0.122 *	0.144	0.164	0.146	0.141	-0.067	-0.032	-0.002
Current smoker	0.137 *	0.075	0.110 *	-0.022	0.005	0.091	0.112	-0.221 ***	-0.206 ***	-0.068
M-HAQ	0.024	0.009	0.000	-0.030	-0.064	-0.024	-0.053	0.112 *	0.032	0.138 *
DAS28-CRP	0.061	0.052	0.077	0.023	-0.009	0.064	0.013	0.135 **	0.101	0.048
SDAI	0.041	0.033	0.067	0.009	-0.024	0.050	-0.006	0.112 **	0.089	0.02
Pain	0.012	-0.004	-0.011	0.029	0.003	0.051	0.043	0.011	0.014	0.124 *
ESR	0.044	0.059	0.076	0.035	0.007	0.089	0.067	0.267 ***	0.196 ***	0.115 *
CRP	0.000	-0.011	0.023	0.014	0.021	0.015	-0.009	0.151 **	0.091	0.091
CRP >8	-0.02	-0.021	0.008	0.002	0.008	-0.015	-0.029	0.091	0.048	0.028
SJC28	0.019	0.010	0.055	-0.019	-0.059	0.036	0.000	0.094	0.084	-0.043
TJC28	0.082	0.080	0.103	0.008	-0.022	0.065	0.005	0.124 *	0.102	0.009
Total SvH score	0.036	0.051	0.049	0.009	0.024	0.026	-0.005	0.491 ***	0.391 ***	0.013
SvH erosion score	0.04	0.035	0.052	0.021	0.049	0.050	0.022	0.360 ***	0.287 ***	0.02

*p <0.05, **p <0.01, ***p <0.001. *Anti-CCP2* antibodies against citrullinated peptides, second generation, *RF* rheumatoid factor, *BMI* body mass index, *M-HAQ* Modified Health Assessment Questionnaire, *DAS28* Disease Activity Score in 28 joints, *SDAI* Simplified Disease Activity Score, *ESR* erythrocyte sedimentation rate, *CRP* C-reactive protein, *SJC* swollen joint count, *TJC* tender joint count, *SvH* progression in the total Sharp score modified by van der Heijde

Univariate analyses of single biomarkers and their combinations were evaluated in relation to total and erosive joint damage progression over 5 years of disease (Table 3). Definite progression of total (ΔSvH ≥5) and erosive joint damage (ΔErosion ≥5) were associated in univariate analyses with the following baseline variables: CRP >8.0 mg/L, age ≥65 years, positive anti-Sa, positive RF, positive anti-CCP2 and 14-3-3η ≥0.50, with the RR ranging from 1.51–2.22 (Table 3). Relative to erosive progression, a slightly weaker RR for total SvH progression was observed with all variables, with the exception that age ≥65 years was more strongly associated with total than erosive progression. Coexistence of both 14-3-3η ≥0.50 and CRP >8.0 increased the RR of erosive progression ≥5 to 3.48 (2.17–5.56), *p* <0.001 relative to the absence of both; combining 14-3-3η ≥0.50 with age ≥65 years increased the RR of erosive progression to 3.46 (2.21–5.42), *p* <0.001. Combining 14-3-3η ≥0.50 with either positive RF, anti-CCP2 or anti-Sa did not markedly improve the RR for erosive progression associated with antibodies (2.27, 2.32 and 2.80, respectively). The optimal univariate combined predictor for erosive progression ≥5 was obtained using a combination of CRP >8.0 mg/L and 14-3-3η ≥0.50 ng/ml and age ≥65 years, with a RR of 5.49 (2.73–11.08) compared to the absence of all three variables (Table 3).

Multivariate predictive models using continuous and dichotomous baseline biomarkers and their significant interactions were also evaluated in relation to definite total and erosive damage progression over 5 years of disease (Table 5). Multivariate GLM analysis with repeated measures of continuous variables showed that baseline age, CRP levels, positive anti-Sa status and 14-3-3η levels were the independent variables significantly associated with both ΔSvH ≥5 and ΔErosion ≥5 over time. In multivariate GEE analysis with repeated measures using dichotomous variables and their interactions, age ≥65 years, CRP >8.0 mg/L and 14-3-3η ≥0.50 ng/ml were the independent predictors of ΔSvH ≥5; age ≥65 years, CRP >8.0 mg/L and 14-3-3η ≥0.50 were again the significant predictors of erosive progression ≥5, together with anti-Sa and RF positivity, and the interaction of 14-3-3η ≥0.50 with antibody positivity (Table 5).

**Table 5:** Multivariate analysis of biomarkers and their interactions to predict radiographic and erosive progression

GLM	ΔSvH estimate (SE)	ΔErosion estimate (SE)
14-3-3η	0.098 (0.045) *	0.088 (0.033) **
Age (years)	0.106 (0.017) ***	0.056 (0.013) ***
Anti-CCP2	0.003 (0.002)	0.001 (0.002)
RF	0.000 (0.001)	0.000 (0.001)
Anti-Sa positive	3.104 (0.747) ***	2.878 (0.543) ***
CRP	0.032 (0.008) ***	0.025 (0.006) ***
GEE	ΔSvH ≥5 RR (95 % CI)	ΔErosion ≥5 RR (95 % CI)
14-3-3η ≥0.50 ng/ml	2.176 (1.193–3.970) *	2.276 (1.448–3.577) ***
Age ≥65 years	2.680 (1.859–3.864) ***	2.751 (1.760–4.301) ***
Anti-CCP2-positive	1.048 (0.696–1.577)	1.134 (0.635–2.025)
RF-positive	1.191 (0.739–1.921)	1.689 (1.042–2.738) *
Anti-Sa-positive	1.537 (0.708–3.335)	2.507 (1.348–4.659) **
CRP >8.0 mg/L	1.425 (1.112–1.826) **	1.834 (1.304–2.578) ***
14-3-3η ≥0.50 * age ≥65 years	0.677 (0.396–1.156)	*Not included*
14-3-3η ≥0.50 * RF-positive	0.724 (0.386–1.358)	0.552 (0.310–0.983) *
14-3-3η ≥0.50 * anti-Sa positive	0.898 (0.470–1.717)	0.400 (0.204–0.786) **
Age ≥65 * anti-CCP2 positive	0.899 (0.507–1.596)	0.692 (0.351–1.365)
Age ≥65 * anti-Sa positive	0.593 (0.345–1.017)	*Not included*
RF-positive * anti-Sa positive	1.256 (0.607–2.597)	*Not included*
Anti-CCP2-positive * anti-Sa positive	*Not included*	0.856 (0.424–1.728)

Age, gender, 14-3-3η, C-reactive protein (*CRP*) and rheumatoid arthritis-associated rheumatoid factor (*RF*), antibodies against citrullinated peptides, second generation (*anti-CCP2*) and anti-Sa antibodies, and all their interaction terms were included in multivariate generalized linear model (*GLM*) or general estimating equation (*GEE*) analysis. Only the models with the lowest Akaike information criterion (AIC) or quasi-AIC are presented. *p <0.05, **p <0.01, ***p <0.001. *SE* standard error *ΔSvH* progression in the total Sharp score modified by van der Heijde, *ΔErosion* progression in the erosion component of the Sharp score modified by van der Heijde. *Not included*variable excluded from the final model

## Discussion

We show that serum 14-3-3η protein levels can be used in addition to baseline RA-associated antibodies to facilitate early identification among EPA patients, of those RA patients likely to have poor outcomes, both clinically and radiographically. 14-3-3η-positive status can thus assist primary care providers during referral of patients to rheumatologists, and may help rapid initiation of a targeted pharmacological intervention. Furthermore, our results show that elevated levels of 14-3-3η can be used in combination with known prognostic biomarkers to identify patients with the worst prognosis for radiographic progression, both initially in untreated patients and in patients already under treatment. A higher level of baseline 14-3-3η (≥0.50 ng/ml) appears superior to the manufacturer-recommended threshold of 0.19 ng/ml to identify those patients most likely to progress rapidly, potentially mandating earlier and more specific antirheumatic strategies.

14-3-3η protein presents mechanistic potential distinct from those of other known RA biomarkers. 14-3-3η is found at increased concentrations in the serum of RA patients, and even more so in their synovial fluid. In vitro and ex vivo, extracellular 14-3-3η acts as a ligand that increases production of inflammatory mediators such as interleukin 6 (IL-6) and tumor necrosis factor α (TNFα), and osteoclast-activating factors such as receptor activator of nuclear factor kappa-B ligand (RANKL). Similar to high or sustained levels of elevated CRP, high baseline and persistently high levels of 14-3-3η are associated with a worse prognosis. Inflammation and joint damage, which are now understood to be processes that uncouple along the course of disease and treatment strategies, have been tightened to achieve both clinical and joint damage remission. CRP and 14-3-3η are both associated with joint damage progression at 5 years and their respective titers are not correlated, consistent with distinct roles in RA disease processes [19]. Surprisingly, combining elevated 14-3-3η with RA-associated antibodies did not markedly improve the RR for radiographic progression found with elevated 14-3-3η or antibodies alone. Positive RF presents similarly high correlations with both elevated 14-3-3η protein (*r* =0.679) and positive anti-CCP2 (*r* =0.661). This does not result from a spurious effect of RF on the performance of the 14-3-3η ELISA assay itself [19]. We postulate that extracellular 14-3-3η may stimulate B cells, potentially leading to or stimulating the production of RA-associated antibodies; further investigation is needed to answer this question. As CRP, RA-associated antibodies and 14-3-3η protein represent modifiable joint damage mechanism markers with very distinct amplitude and kinetics of response to specific treatments, the complementary information provided by all three may enhance clinical management strategies [20]. Univariate and multivariate interaction analyses further revealed that combining 14-3-3η and CRP resulted in better predictors of future radiographic damage than either marker alone. Concomitant serial testing of the modifiable CRP and 14-3-3η markers may thus assist with tight-control RA treatment strategies. As increased progression persisted in patients who were 14-3-3η-positive despite being in SDAI remission, innovative therapies may be needed to further improve the final outcomes of these patients.

Our results show increasing age as an important predictor of joint damage progression, especially of joint space narrowing. The reasons for the detrimental impact of age are many, including increased susceptibility of osteoarthritis-damaged cartilage to degradative enzymes induced by inflammation, the effect of ongoing osteoarthritis itself superimposed on that of inflammatory arthritis, greater susceptibility of aging bone to resorption by inflammation-activated osteoclasts, and the limitations in choices of treatment used to control arthritis in this fragile population. As older patients are particularly susceptible to joint damage in the presence of elevated 14-3-3η and CRP, this combination of unfavorable biomarkers might be used to identify those patients most likely to benefit from more targeted treatment strategies.

Our study has numerous strengths. First we followed a large number of patients over a 5-year period following onset of disease. Second, the assessment of the patients was exhaustive, including serial sera and SvH scores on radiographs of the hands and feet. Third, the prospective nature of our study allowed us to determine the relative contribution of a number of baseline and early variables to disease activity and radiographic progression observed longitudinally. Fourth, our patients were treated-to-target rapidly after symptom onset, similar to currently recommended strategies. Fifth, our patients had minimal missing data, and no imputations were done. Sixth, our patients originate from a cohort of consecutive EPA patients, with minimal bias and variability of evaluation and treatment. Nonetheless, the vast majority (>90 %) already fulfilled classification criteria for RA at baseline.

Our study also has limitations. First, only those EPA patients followed for 5 years were selected for this study. Indeed, the selected patients were significantly younger (60 vs 64.5 years; *p* = 0.01), reported more pain at baseline (mean visual analog scale 0-100 (VAS) score 59.0 vs 51.5; *p* = 0.007), and were more frequently RF-positive (44.1 % vs 35.7 %. *p* = 0.02) and anti-CCP2-positive (40.0 % vs 30.7 %, *p* = 0.01) but not anti-Sa/citrullinated vimentin positive (22.1 % vs 17.2 %, *p* = 0.112) than the other patients from our cohort, but did not differ in any other significant variable. These small differences are unlikely to affect the applicability of our results to most EPA patients. Second, the treatment prescribed for patients was not uniform but remained largely the same for patients with similar disease activity, as it was selected to rapidly attain a state of zero swollen joints, with minimal long-term corticosteroids. This may better reflect current clinical practice, however. Third, we used a higher threshold than the upper normal limit recommended by the manufacturer of the assay (≥0.50 vs ≥0.19 ng/ml). Like higher titers of antibodies, higher levels of 14-3-3η (≥0.50 ng/ml) present better prognostic properties. The threshold to be used should thus be determined by the context in which the assay is performed: when screening EPA patients for referral, a level of ≥0.19 ng/ml might be used [19]; for estimation of individual prognosis in order to define each patient’s individual treatment strategy, a threshold ≥0.50 ng/ml might be recommended. Fourth, the optimal use of currently available biomarkers to predict poor outcomes will require a thorough examination in multiple different cohorts. Fifth and most important, we observed that positive 14-3-3η levels identified patients at higher risk of worse outcomes; it remains to be determined whether using the results of the assay to refine optimized treatment can prevent or delay the development of poor outcomes.

## Conclusion

Measuring serum levels of the 14-3-3η protein appears to be a positive addition to guide early identification of EPA patients in need of an urgent referral to rheumatologists. Moreover, the presence of elevated levels of 14-3-3η protein clearly identifies a subset of patients at baseline with a high risk of clinically refractory disease and significant joint damage over the next 5 years. In addition, the persistence of a high level of 14-3-3η protein despite treatment identifies patients at high risk of structural damage, even in patients achieving SDAI remission. And finally, high CRP and elevated 14-3-3η protein at baseline and under treatment represent an adverse prognostic signature suggesting that a majority of these patients will deteriorate significantly, especially in older individuals. Corroboration of our results in other cohorts with different genetic backgrounds and treatment strategies will establish the role that measurement of 14-3-3η protein will play in the diagnosis of RA among EPA patients and in the development of a personalized medicine approach in EPA.

## Additional files

Additional file 1: Table S1. Distribution of 14-3-3η over time according to baseline positivity. (PDF 167 kb) Additional file 2: Figure S1. Simplified Disease Activity Index (*SDAI*) remission (**a**) and radiographic progression (**b**) over 5 years according to baseline 14-3-3η positivity set at ≥0.19 ng/ml. (PDF 391 kb)

## Abbreviations

Ab(Abs): Autoantobody(ies)
ACR: American College of Rheumatology
AIC: Akaike information criterion
anti-CCP2: antibodies to cyclic citrullinated peptides, second generation
anti-Sa: antibodies to Sa/citrullinated vimentin
AUC: area under the curve
CHUS: Centre hospitalier universitaire de Sherbrooke
BMI: body mass index
CI: confidence interval
CRP: C-reactive protein
DAS28: Disease Activity Score in 28 joints
DAS28-CRP: Disease Activity Score using 28 joints and C-reactive protein
DMARD: disease-modifying antirheumatic drug
ELISA: enzyme-linked immunosorbent assay
EPA: recent-onset (early) inflammatory polyarthritis
ESR: erythrocyte sedimentation rate
EULAR: European League Against Rheumatism
EUPA: early undifferentiated polyarthritis
GEE: generalized estimating equation
GLM: generalized linear model
IL: interleukin
M-HAQ: Modified Health Assessment Questionnaire
IQR: interquartile range
MTX: methotrexate
QIC: quasi-Aikake information criteria
RA: rheumatoid arthritis
RANKL: receptor activator of nuclear factor kappa-B ligand
RF: rheumatoid factor
RR: relative risk
ROC: receiver operating characteristic
SDAI: Simple Disease Activity Score
SD: standard deviation
SDC: smallest detectable change
SEM: standard error of the mean
SJC: swollen joint count
SvH: Sharp score modified by van der Heijde
T2T: treat-to-target
TJC: tender joint count
TNF: tumor necrosis factor
VAS: visual analog scale 0-100
ΔErosion: progression in the erosion component of the Sharp score modified by van der Heijde
ΔSvH: progression in the total Sharp score modified by van der Heijde
