# Supplementary material for: Serum levels of 14-3-3η protein supplement C-reactive protein and rheumatoid arthritis-associated antibodies to predict clinical and radiographic outcomes in a prospective cohort of patients with recent-onset inflammatory polyarthritis
Source: Arthritis Res Ther. 2016 Feb 1;18:37. doi: 10.1186/s13075-016-0935-z (PMC4736641; doi:10.1186/s13075-016-0935-z)
Supplement: Additional file 1: Table S1. — Distribution of 14-3-3η over time according to baseline positivity. (PDF 167 kb) [file 13075_2016_935_MOESM1_ESM.pdf]

Supplementary Table 1. Distribution of 14-3-3η over time according to baseline positivity

	Baseline	18 months	30 months	42 months	60 months
**All patients:**					
n total	331	324	319	323	323
14-3-3η, median (IQR)	0.14 (0.03-1.86)	0.11 (0.03-1.12)	0.11 (0.03-0.99)	0.10 (0.02-0.93)	0.13 (0.02-1.14)
14-3-3η≥0.19, n (%)	153 (46.2)	149 (46.0)	134 (42.0)	127 (39.3)	143 (44.3)
14-3-3η≥0.50, n (%)	119 (36.0)	105 (32.4)	101 (31.7)	93 (28.8)	108 (33.4)
**Patients with 14-3-3η≥0.19 at baseline:**					
n total	153	151	148	148	149
14-3-3η, median (IQR)	2.79 (0.59-21.00)	1.27 (0.31-9.01)	1.03 (0.24-11.94)	1.08 (0.24-7.83)	1.13 (0.28-8.12)
14-3-3η≥0.19, n (%)	---	130 (86.1)	120 (81.1)	117 (79.1)	124 (83.2)
**Patients with 14-3-3η≥0.50 at baseline:**					
n total	119	117	114	116	118
14-3-3η, median (IQR)	6.26 (1.59-21.00)	2.27 (0.69-14.12)	1.98 (0.53-15.63)	2.15 (0.39-12.79)	2.28 (0.52-13.19)
14-3-3η≥0.50, n (%)	---	94 (80.3)	89 (78.1)	82 (70.7)	89 (75.4)
**Patients with 14-3-3η<0.19 at baseline:**					
n total	178	173	171	175	174
14-3-3η, median (IQR)	0.04 (0.01-0.09)	0.03 (0.01-0.08)	0.03 (0.01-0.08)	0.03 (0.01-0.07)	0.03 (0.01-0.08)
14-3-3η<0.19, n (%)	---	154 (89.0)	157 (91.8)	165 (94.3)	155 (89.1)
**Patients with 14-3-3η<0.50 at baseline:**					
n total	212	207	205	207	205
14-3-3η, median (IQR)	0.06 (0.02-0.12)	0.05 (0.02-0.10)	0.05 (0.01-0.12)	0.04 (0.01-0.10)	0.04 (0.01-0.14)
14-3-3η<0.50, n (%)	---	196 (94.7)	193 (94.1)	196 (94.7)	186 (90.7)

IQR: Interquartile range (25th-75th percentiles)
