# Supplementary material for: Serum levels of 14-3-3η protein supplement C-reactive protein and rheumatoid arthritis-associated antibodies to predict clinical and radiographic outcomes in a prospective cohort of patients with recent-onset inflammatory polyarthritis
Source: Arthritis Res Ther. 2016 Feb 1;18:37. doi: 10.1186/s13075-016-0935-z (PMC4736641; doi:10.1186/s13075-016-0935-z)
Supplement: Additional file 2: Figure S1. — Simplified Disease Activity Index (SDAI) remission (a) and radiographic progression (b) over 5 years according to baseline 14-3-3η positivity set at ≥0.19 ng/ml. (PDF 391 kb) [file 13075_2016_935_MOESM2_ESM.pdf]

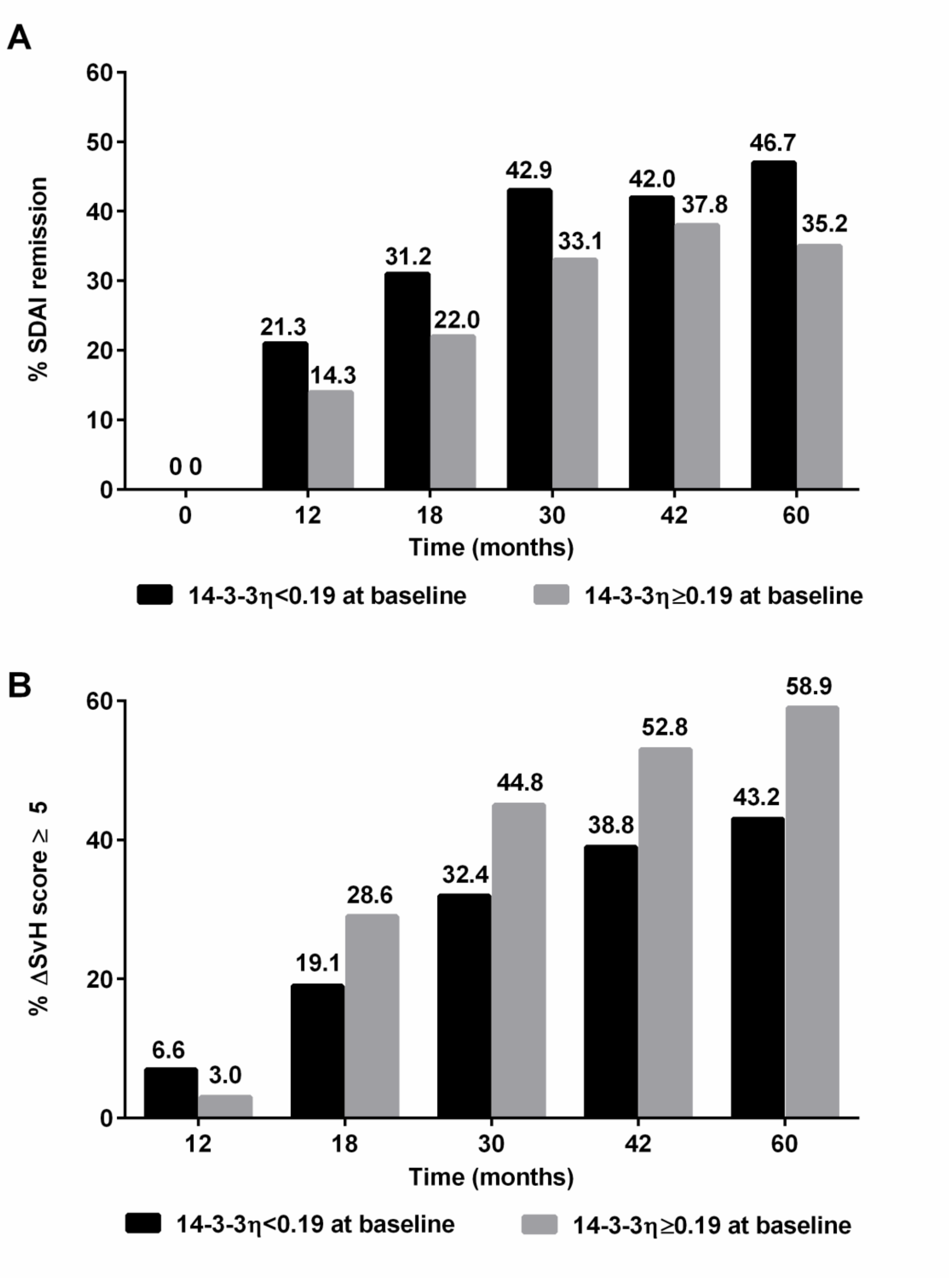

Supplementary Figure 1. SDAI remission (A) and radiographic progression (B) over 5 years according to baseline 14-3-3η positivity. GEE analyses were performed to compare SDAI scores and radiographic progression over time with baseline 14-3-3η ≥0.19 ng/ml. A) SDAI remission over time was significantly lower in patients with baseline 14-3-3η ≥0.19 (RR=0.79 (95%CI 0.64-0.97), p=0.03) B) Definite radiographic progression (ΔSvH ≥5) over time was significantly higher in patients with baseline 14-3-3η ≥0.19 ng/ml (RR=1.39 (95%CI 1.11-1.74), p=0.005).
